# Novel Computational and Artificial Intelligence Models in Cancer Research

**DOI:** 10.3390/cancers17010116

**Published:** 2025-01-02

**Authors:** Li Liu, Fuhai Li, Xiaoming Liu, Kai Wang, Zhongming Zhao

**Affiliations:** 1 College of Health Solutions, Arizona State University, Phoenix, AZ 85004, USA; 2 Biodesign Institute, Arizona State University, Tempe, AZ 85281, USA; 3 Institute for Informatics, Data Science and Biostatistics, Washington University in St. Louis, St. Louis, MO 63108, USA; fuhai.li@wustl.edu; 4 Department of Pediatrics, Washington University in St. Louis, St. Louis, MO 63108, USA; 5 University of South Florida Genomics & College of Public Health, University of South Florida, Tampa, FL 33612, USA; xiaomingliu@usf.edu; 6 Raymond G. Perelman Center for Cellular and Molecular Therapeutics, Children’s Hospital of Philadelphia, Philadelphia, PA 19104, USA; wangk@chop.edu; 7 Department of Pathology and Laboratory Medicine, University of Pennsylvania, Philadelphia, PA 19104, USA; 8 Center for Precision Health, McWilliams School of Biomedical Informatics, The University of Texas Health Science Center at Houston, Houston, TX 77030, USA

## 1. Introduction

The ICIBM 2023 marked the 11th annual conference of its kind, with the ICIBM recently becoming the official conference of the International Association for Intelligent Biology and Medicine (IAIBM), showcasing cutting-edge advancements at the intersection of computation and biomedical research. A summary of the ICIBM 2023 program is provided by Li et al. [1]. As in previous years, cancer research remained a prominent focus, with numerous presentations highlighting innovative approaches to understanding and treating this complex disease. To summarize these contributions, selected works have been compiled in this Special Issue, “Novel Computational and Artificial Intelligence Models in Cancer Research”, reflecting the significant progress and interest in this critical area of study.

## 2. Summary of Studies

### 2.1. Cancer Imaging

AI advances in cancer imaging research leverage computer vision technologies, one of the earliest domains where machine learning and AI methods were successfully applied [2]. Medical imaging data, rich in detailed structural and functional information, naturally lend themselves to AI’s ability to extract patterns, enhance predictions, and support clinical decision-making [3,4]. Specifically in the context of cancer, AI demonstrates the ability to automate the initial interpretation of medical images, improve radiographic detection, and support clinical decision-making [5]. Two studies in this Special Issue demonstrate the transformative potential of AI when applied to imaging data.

The study by Hu et al. introduces a composite neural network within a federated learning environment that integrates multisource multimodal data to diagnose lymph node metastasis [6]. This approach jointly optimizes local models using data from individual hospitals and leverages a global model to aggregate and enhance the performance of local predictions. Applied to patients who have gynecologic malignancies and undergo preoperative imaging assessments, the model achieves remarkable diagnostic performance by integrating magnetic resonance imaging (MRI) data and clinical text data. The federated learning framework also addresses privacy concerns by ensuring local data protection and confidentiality to facilitate multi-institutional collaboration.

The study by Das et al. presents a Bayesian framework designed to quantify glioblastoma growth based on multimodal MRI data [7]. This approach extracts the current tumor volume and various radiomic parameters from an MRI scan, and integrates these features with spatial information and glioma diffusion properties within a structural equation model to predict the ultimate tumor volume, i.e., the number of cells that could proliferate from this tumor during its survival time. When tested on preoperative baseline multimodal MRI scans of glioblastomas, this approach demonstrates reliable predictions of the growth of fourth-grade tumors using a time-independent approach.

### 2.2. Molecular Pathways and Drivers of Cancer

Cancer has molecular underpinnings at various levels, including genetic mutations, epigenetic changes, transcriptional dysregulation, altered protein–protein interactions, and impaired microenvironment dynamics [8,9]. These interconnected processes form complex pathways that are challenging to decipher. Omics studies of bulk tissues and single cells have become powerful tools to investigate these intricate mechanisms [10,11]. However, the high-dimensional and multifaceted nature of omics data makes their interpretation a significant analytical challenge. AI techniques have demonstrated remarkable success in overcoming these challenges, revealing hidden patterns, and identifying actionable targets [12,13,14]. In this Special Issue, five studies present novel AI-driven methods to identify the molecular pathways and drivers of cancer.

Young et al. introduce a redundant-input neural network (RINN) to evaluate the impact of somatic genomic alterations on cancer cell signaling [15]. Designed as a causal graphical model, RINN traces the flow of information from genomic alterations to signaling proteins and pathways, ultimately linking these changes to differential gene expression. By training the model on TCGA pan-cancer data, the authors identify instances where multiple somatic genomic alterations converge on and perturb the same signaling pathways, highlighting the shared functional impacts of diverse genomic changes in cancer.

Yang et al. introduce MultiFDRnet, an algorithm designed to detect perturbed subnetworks by aggregating somatically mutated genes across multiple protein–protein interaction networks [16]. MultiFDRnet integrates networks from different databases and tissue types, leveraging the complementary strengths of diverse network annotations while maintaining control over the overall false discovery rate. The method is extensively validated using both simulated and real cancer data. The application of MultiFDRnet to bladder cancer and head and neck cancer samples reveal hotspots in generic pathways shared across cancers as well as cancer-type-specific pathways.

Wei et al. present an immune-related risk score model for endometrial cancer, integrating clinical and molecular data to stratify patient outcomes [17]. The workflow begins with constructing a gene co-expression network, from which immune-related modules are mined to identify genes associated with overall survival. These genes are then linearly combined to generate a risk score. Beyond serving as a prognostic marker, further analysis of the predictor genes’ expression levels reveals their associations with the molecular basis of the disease, tumor immune microenvironment, and responses to treatments.

Zhang et al. introduce scGEM, a nested tree-structured nonparametric Bayesian model designed to construct gene co-expression modules (GEMs) from single-cell transcriptomic data [18]. This algorithm models the hierarchical relationships among cells, capturing the gradual changes in both the topology of GEMs and the transcription abundance of their member genes during cellular differentiation and specialization. The resulting GEMs enhance the deconvolution of functional signals in bulk transcriptomic data. Applying scGEM to triple-negative breast cancer data reveals significant correlations between cell-type-specific GEMs and key processes such as lymphocyte infiltration and the cell cycle, both of which are critical in tumorigenesis.

Chen et al. focus on identifying cancer drivers in non-human model organisms [19]. They employ transfer learning techniques to adapt the GUST model [20], originally developed to distinguish oncogenes and tumor suppressor genes in human tumors, to mouse models. By comparing genetic drivers in humans to those in mice across different cancer types, the study identifies both conserved and unique tumorigenesis mechanisms across species and cancer types. This approach bridges the translational gap between human and murine cancer research, providing a robust framework for better aligning non-human tumor models with human cancer studies.

### 2.3. Benchmarking Computational Tools

With the rapid evolution of precision oncology, multiple competing methods and models often emerge for the same task, reflecting the dynamic nature of innovation and discovery [21,22]. While this diversity fuels progress, it also underscores the need for rigorous evaluation to ensure reliability, reproducibility, and meaningful comparisons across tools. Benchmarking provides a standardized framework for assessing the strengths and limitations of various methods, facilitating their refinement and ensuring their applicability in diverse clinical and research settings [23]. Two studies in this issue exemplify the importance of benchmarking.

The first study focuses on evaluating transcriptomic biomarkers for immune checkpoint blockades (ICBs) [24]. Kang et al. manually curate and uniformly process transcriptomic data from over 1400 cancer patients, integrating these data with clinical data. Using this extensive dataset, they systematically assess the performance of 39 biomarker sets in predicting ICB responses and survival outcomes across various contexts. Their analysis reveals that while most biomarkers exhibit low stability and robustness across datasets, two biomarker sets demonstrate strong correlations with ICB responses, and another two are significantly associated with clinical outcomes. An online resource, ICB-Portal, hosts the datasets and evaluation results from this study, enabling other researchers to test and refine their custom biomarkers.

The second study assesses data quality in small RNA profiling studies of human extracellular vesicles (EVs) [25]. Wang et al. reanalyze 83 small RNA expression datasets, encompassing samples from diverse sources, representing varying phenotypes, and prepared using different techniques. When evaluated on a comprehensive set of quality metrics, these datasets exhibit significant biases associated with technical platforms, RNA composition, and RNA biotype enrichment. These biases contribute to substantial variability that can obscure true biological signals and compromise reproducibility. While the findings offer valuable guidance for quality control, EV isolation methods, and data interpretation, they also highlight the urgent need for standardized protocols in EV research to improve data consistency and ensure the reliability of biological conclusions in future studies.

## 3. Conclusions

The nine studies featured in this Special Issue exemplify the transformative potential of computational and AI-driven methods in cancer research. From developing novel models to benchmarking existing tools, these contributions address key challenges in oncology and medical imaging and pave the way for innovations in diagnosis, treatment, and prevention. By fostering collaboration between computational scientists, biologists, and clinicians, this Special Issue reflects the interdisciplinary nature of modern cancer research. We hope it inspires further exploration and application of AI technologies, driving progress toward a future of precision oncology.
